# The Effects of a Health Care Chatbot’s Complexity and Persona on User Trust, Perceived Usability, and Effectiveness: Mixed Methods Study

**DOI:** 10.2196/41017

**Published:** 2023-02-01

**Authors:** Joshua Biro, Courtney Linder, David Neyens

**Affiliations:** 1 Department of Industrial Engineering Clemson University Clemson, SC United States

**Keywords:** electronic health record, EHR, health information, health education, patient education, chatbot, virtual agent, virtual assistant, usability, trust, adoption, artificial intelligence, effectiveness

## Abstract

**Background:**

The rising adoption of telehealth provides new opportunities for more effective and equitable health care information mediums. The ability of chatbots to provide a conversational, personal, and comprehendible avenue for learning about health care information make them a promising tool for addressing health care inequity as health care trends continue toward web-based and remote processes. Although chatbots have been studied in the health care domain for their efficacy for smoking cessation, diet recommendation, and other assistive applications, few studies have examined how specific design characteristics influence the effectiveness of chatbots in providing health information.

**Objective:**

Our objective was to investigate the influence of different design considerations on the effectiveness of an educational health care chatbot.

**Methods:**

A 2×3 between-subjects study was performed with 2 independent variables: a chatbot’s complexity of responses (eg, technical or nontechnical language) and the presented qualifications of the chatbot’s persona (eg, doctor, nurse, or nursing student). Regression models were used to evaluate the impact of these variables on 3 outcome measures: effectiveness, usability, and trust. A qualitative transcript review was also done to review how participants engaged with the chatbot.

**Results:**

Analysis of 71 participants found that participants who received technical language responses were significantly more likely to be in the high effectiveness group, which had higher improvements in test scores (odds ratio [OR] 2.73, 95% CI 1.05-7.41; *P*=.04). Participants with higher health literacy (OR 2.04, 95% CI 1.11-4.00, *P*=.03) were significantly more likely to trust the chatbot. The participants engaged with the chatbot in a variety of ways, with some taking a conversational approach and others treating the chatbot more like a search engine.

**Conclusions:**

Given their increasing popularity, it is vital that we consider how chatbots are designed and implemented. This study showed that factors such as chatbots’ persona and language complexity are two design considerations that influence the ability of chatbots to successfully provide health care information.

## Introduction

As health care technology advances, internet usage increases, and cultural norms shift (eg, in response to the COVID-19 pandemic), people are receiving more health care information from virtual mediums (eg, telehealth) than ever before [1]. This rising adoption of telehealth provides new opportunities for more effective and equitable health care information mediums. One such promising health care information medium is chatbots. Chatbots provide a conversational, personal, and comprehendible avenue for learning about health care information. The conversational aspect of chatbots has been shown to help support people in online groups for various health conditions [2]. The personal aspect of chatbots has been shown to excel at providing information on sensitive topics, such as sex-, drug-, and alcohol-related questions of young adults, as chatbots are perceived to be faster and more anonymous than conventional search engines for discussing these sensitive issues without judgment [3]. The comprehendible aspect of chatbots is perhaps their greatest asset for health care applications, as chatbots have been shown to be a more effective resource for finding health care information than conventional internet-based searching for individuals with low health literacy [4]. Health literacy is crucial for empowering people to manage their health [5], yet most health information is written at levels that exceed people’s understanding [6]. This disconnect between health literacy and health information is estimated to cost the United States’ health care system between US $106 billion and US $238 billion annually [7,8]. Low health literacy has been shown to be associated with various poor health outcomes (eg, more hospitalization and higher mortality rate) and poorer use of health care services (eg, poorer ability to interpret health messages and take medications appropriately) [9]. People with low health literacy have different approaches to learning health information; lower health literacy is associated with higher use and more trust in health information from television, social media, blogs, or celebrity web pages as well as lower use of medical websites and less trust in health information from specialist doctors [10]. About 35% of the US population has only a basic or below basic health knowledge and is disproportionately represented by low-income or ethnic minority populations [11]. The ability of chatbots to provide comprehendible information to those with lower health literacy is one potential remedy for this unequitable health information disconnect.

The potential benefits that chatbots can provide have led to their implementation in a variety of health care contexts, including diet recommendations [12], smoking cessation [13], and cognitive behavior therapy [14], but more research needs to be done to understand how chatbots should be designed to be most effective. In a retail setting, it has been shown that a chatbot’s language and communication style influences ease of use and engagement [15]. However, users interacting with health care information chatbots may have different needs and expectations than when interacting with chatbots in other industries, and there is little research investigating the influence of design considerations of chatbots on their effectiveness for providing health care information. As chatbots have a history of being biased and unfair [16,17], efforts to explore design considerations of chatbots must account for the intersectionality of identities and be considerate of all people. A potential avenue for helping users connect with chatbots is to give the chatbot an identity or persona. It has been shown that other virtual agents may be more or less effective due to their perceived character [18], yet the effect that different personas have on the effectiveness of a health care information chatbot is unclear. Thus, the primary objective of this study was to examine the effects of an educational health care chatbot, as it differs in complexity of responses (technical vs nontechnical language) and the presented qualifications of its persona (eg, Doctor, Nurse, or Nursing Student persona) on perceived usability, trust, and effectiveness. The secondary objective was to identify similarities and differences in how users conversed with the chatbot.

## Methods

### Study Design

In this study, participants were tasked with interacting with the chatbot to seek information about blood pressure. The experiment was a 2×3 between-subjects design, in which the chatbot with which the participants interacted differed in the complexity of its responses (either technical or nontechnical language) and the presented qualifications of its persona (either Doctor, Nurse, or Nursing Student).

### Ethics Approval

This study was reviewed and approved by the Clemson University Institutional Review Board (IRB2019-411).

### Chatbot Design

The most common purpose of chatbots in health care has been to provide education and training for conditions (eg, mental health, type 2 diabetes, breast cancer, hypertension, asthma, pain monitoring, and language impairment) [19]. To emulate this common purpose, the chatbot created in this study was designed to answer questions and provide general health information about blood pressure. The chatbot used in this research emulated a pattern-matching chatbot rather than one which uses artificial intelligence. Pattern matching occurs when the question patterns match certain answer patterns. For this study, we created predefined answers that offered the same information in either technical or nontechnical language. The experimenter delivered the chatbot responses to questions asked by the participant using a “Wizard of Oz” technique. In this type of experiment, a participant interacts with a system that they expect to be autonomous but is secretly controlled by a member of the research team [20-22]. A prepopulated response list to possible participants’ questions was created, evaluated, and refined through pilot testing. The responses were created to address all questions that pilot tests identified as well as other possible generic question responses. These generic responses accounted for unanticipated questions or off-topic discussions not related to blood pressure. The generic responses did not change between technical and nontechnical conditions. An example of a generic response is, “I am sorry, I am unable to answer that question. Do you have another question about blood pressure?” An intensive care unit nurse was consulted to verify our chatbot content and to identify any additional information we may have missed or that was outdated or incorrect.

To differentiate between the complexity of the responses (technical vs nontechnical), we assessed the reading difficulty of each chatbot response using the Microsoft Word Reading Assessment feature. This feature uses the Flesch-Kincaid readability test, which determines a text’s Flesch reading ease and its Flesch-Kincaid grade level. The Flesch-Kincaid assessments have been used to assess technical manuals, legal documents, and insurance policies [23,24]. The nontechnical responses all had high reading ease and a reading grade level of 8 or below, whereas the technical responses had low reading ease and grade levels of 12 or higher. These reading grade levels were chosen because patient education materials have been found to have mean reading grade levels around 11-14, whereas recommendations for appropriate reading grade levels are 6-8 [25]. Although one possibility to increase the reading level of a response could have been to add additional text or information, this was not done to ensure consistency in the amount of information presented by the chatbot to the participants between technical and nontechnical responses.

The persona that the chatbot represented consisted of 3 possible naming structures (ie, Doctor, Nurse, or Nursing Student). Each of the chatbot personas were named Sarah with only the salutation changing between the conditions (eg, “Dr Sarah,” “Nurse Sarah,” or “Nursing Student Sarah”). This was done to avoid any implicit bias in the persona based on using different names. Each of the personas introduced themselves at the start of the chatbot engagement. For example, “Hello, my name is Dr Sarah. I’m here to help you learn about blood pressure today. You can ask questions about understanding blood pressure, learning how to manage or prevent high blood pressure, who is affected, and more. What is your first question?” Following the initial engagement, the persona identifier was used as an identifier in each response to the participant.

### Participants

Participants were recruited from Clemson university; they were required to be between the ages of 18 and 26 years and to be able to read, write, and speak in English. Participants received a compensation of US $10 for 30 minutes of their time at the end of the session. Participants between the ages of 18 and 26 years were chosen so that the participant population likely had a similar (nominal) level of knowledge about blood pressure.

### Procedure

Following informed consent procedures, participants completed a demographic survey and then an experimenter assessed the participants’ health literacy using the Short Assessment of Health Literacy—English [26]. Participants then completed a multiple-choice test on blood pressure topics (henceforth referred to as the “pretest”). The blood pressure topics included the effects of high and low blood pressure, factors associated with blood pressure issues, and risk factors for high blood pressure. These factors were included based on the content in health textbooks and web-based resources that discuss blood pressure, common questions, and common misconceptions [27-30]. After the pretest, participants were instructed on how to begin using the chatbot and were informed that they had up to 15 minutes to learn about blood pressure by interacting with the chatbot. The experimenter, stationed in a separate room from the participant, ran the chatbot using a Wizard of Oz type of structure (ie, they responded to the participants’ questions with preconstructed answers). After interacting with the chatbot, participants took the same multiple-choice test on blood pressure topics (henceforth known as the “posttest”). Following the posttest, participants were given the Post‐Study System Usability Questionnaire (PSSUQ) [31] and a survey assessing the trustworthiness, credibility, and perceived ease of use of the chatbot [32].

### Analysis

Participants’ perceived usability of the chatbot was measured via the PSSUQ [31] and was evaluated using a linear regression model. Participant’s trust in the chatbot was measured via a question assessing how much the participant agreed with the statement “I trust the chatbot” on a 7-point Likert scale (“strongly disagree” to “strongly agree”). This Likert scale was converted to a binary variable representing those who trusted the chatbot (ie, participants that responded with “somewhat agree,” “agree,” and “strongly agree”) and those who did not trust the chatbot (ie, all other responses). Trust was evaluated using a binary logistic regression model. The chatbot’s effectiveness was operationalized as the difference in pretest versus posttest scores from the blood pressure knowledge test. Effectiveness was evaluated using a median split binary logistic regression model. All regression models started by including response complexity and chatbot persona as well as the following demographic variables: self-identified gender, health literacy, ethnicity, and student status (eg, graduate or undergraduate student). Demographic variables were removed from the model stepwise following Akaike information criterion minimization until a final model was reached. Additionally, a qualitative transcript review of the participants’ conversation with the chatbot was conducted.

## Results

### Descriptive Statistics

Initially, 74 students participated in the study; however, 3 participant’s data were removed from the data analysis—two due to incomplete data collection and one because the participant did not engage in the task (eg, not asking blood pressure–related questions throughout the experiment). Of the remaining 71 participants, 43 (60.6%) self-identified as female, 30 (42.3%) were graduate students, and 41 (57.7%) were undergraduate students. The average age of the participants was 21.87 (SD 2.58) years. The demographic results are presented in Table 1.

**Table 1:** Characteristics of study participants (N=74).

Variables			Values
Age (years), mean (SD)			21.87 (2.58)
**Gender, n (%)**			
	Male	28 (39.4)	
	Female	43 (60.6)	
**Race, n (%)**			
	Caucasian	49 (69)	
	African American	8 (11.3)	
	Asian	14 (19.7)	
**Student status, n (%)**			
	Undergraduate	41 (57.7)	
	Graduate	30 (42.3)	

### Usability

The average usability score was relatively high (mean 6.00, SD 0.63), indicating high perceived usability of the system. A linear model was constructed to model the usability scores from the independent factors and resulted in residuals that were significantly skewed (Shapiro-Wilk test: skewness 0.959; *P*=.02). Therefore, the PSSUQ average scores were transformed using a square transformation, resulting in a model with residuals that were identified as not being significantly skewed (skewness 0.976; *P*=.18). The linear regression model (Table 2) revealed that participants who self-identified as males (*P*=.049) and participants who interacted with the “Nursing Student” persona of the chatbot (*P*=.02) were significantly more likely to report the chatbot as having a lower usability. Participants who were undergraduate students were significantly more likely to report the chatbot as having a higher usability (*P*=.03).

**Table 2:** Linear regression model predicting usability of the chatbot.

Coefficients	Estimate	SE	*P* value
Intercept	39.5	1.98	<.001
Response complexity (technical language)	–2.34	1.59	.15
Chatbot persona (“Doctor”)	–3.32	1.97	.10
Chatbot persona (“Nursing Student”)	–4.52	1.96	.02
Gender (male)	–3.38	1.70	.049
Student status (undergraduate)	7.05	2.27	.03

### Trust

Only 9 of 71 (12.7%) participants reported not trusting the chatbot. A binary logistic regression model predicting trust (Table 3) revealed that participants with higher health literacy were significantly more likely to trust the chatbot (OR 2.04, 95% CI 1.11-4.00; *P*=.03). No other factors significantly impacted the reported trust in the chatbot.

**Table 3:** Binary logistic regression model predicting trust in the chatbot.

Coefficients	OR^a^ (95% CI)	*P* value
Intercept	<0.001 (<0.001-1.51)	.07
Response complexity (technical language)	0.80 (0.17-3.58)	.77
Chatbot persona (“Doctor”)	0.86 (0.14-4.95)	.87
Chatbot persona (“Nursing Student”)	1.94 (0.27-17.8)	.52
Health literacy score	2.04 (1.11-4.00)	.03

^a^OR: odds ratio.

### Effectiveness

The median difference in pretest versus posttest scores was an improvement of 4 questions, and thus, a median split separated participants who had an improvement of 4 or more into a “high effectiveness” group (n=37) and those who had an improvement less than 4 into a “low effectiveness” group (n=34). A binary logistic regression predicting effectiveness (Table 4) revealed that participants who received technical language responses were significantly more likely to be in the high effectiveness group (OR 2.73, 95% CI 1.05-7.41; *P*=.04) when compared to participants who received nontechnical language responses. No other factors significantly impacted the effectiveness of the chatbot.

**Table 4:** Binary logistic regression model predicting effectiveness of the chatbot.

Coefficients	OR^a^ (95% CI)	*P* value
Intercept	0.87 (0.33-2.25)	.76
Response complexity (technical language)	2.73 (1.05-7.41)	.04
Chatbot persona (“Doctor”)	0.84 (0.25-2.72)	.76
Chatbot persona (“Nursing Student”)	0.52 (0.15-1.69)	.28

^a^OR: odds ratio.

### Qualitative Transcript Review

Analysis of the chatbot conversation transcripts reveals that all of the 71 participants followed the general knowledge–seeking task. However, there were elements of how participants interacted with the chatbot that varied. Only about half of the participants (35/71, 49.3%) asked at least one question using the singular “I” form, often concerning prevention for themselves (ie, “How can I prevent high blood pressure from occurring?”). Of these participants, most (25/35, 71.4%) asked more than one question using the singular “I” form. Generally, the “I” questions could be answered with generic responses, but occasionally participants would ask questions such as “Am I at risk?” which the chatbot, based on the current chatbot pattern matching structure, was not able to answer explicitly for each participant. Only one participant asked the chatbot about assisting others: “How can I help someone with high blood pressure?” When participants received an “I don’t know” response from the chatbot, they generally reverted back to general knowledge seeking with questions like “What is blood pressure?” or “Who is affected most?”

A handful of participants (5/71, 7%) used scenarios at some point in their dialogue to learn about specific factors that could put them at risk of high blood pressure. The scenarios were generally self-centric, in that the participants wanted to know if their specific life circumstances or choices could affect their blood pressure. Textbox 1 summarizes the scenario style questions from the transcripts that demonstrate these scenarios or concerns.

Additionally, the way in which participants interacted with the chatbot’s persona (Doctor, Nurse, or Nursing Student Sarah) varied. When participants initially entered the chatbot, they received a welcome message from Sarah. Only 4 of 71 (5.6%) participants responded with a greeting or addressed Sarah personally (eg, “Hello Nursing Student Sarah, what a strange name. I am Graduate Student (redacted),” or “Hi Sarah!”). An additional person thanked Sarah at one point in their session (“Thanks for helping me Nurse Sarah”), while another two participants just said “Thanks” at the very end of the session. Two of the participants that addressed Sarah at the beginning also either addressed her again in the session or had generic conversation-like comments (eg, “You too, Nursing Student Sarah”). Still other participants said things like “Interesting,” “Okay,” and “That’s scary” when finding out information they did not know or by which they were fascinated.

The way the participants used grammar or shorthand in their conversation with the chatbot was evaluated. Most participants asked their questions using a format similar to “What is high blood pressure?” although even those varied greatly in terms of grammar. Some participants used capitalization and question marks whereas others did not. Other participants preferred statements like “how to prevent blood pressure,” “symptoms of high blood pressure,” and even one as simple as “high blood pressure.” Overall, the way participants formatted their questions grammatically and how they expected to be able to input text and receive corresponding information varied widely, which suggests multiple means of interaction with the chatbot, either as a chatbot conversationally or emulating a search engine.

**Scenario quotes from chatbot transcripts.**

**Quotes**

- I am 25 year old [sic] and my mother and father both have high blood pressure. What are the odds that I get high blood pressure?
- What if I work out but eat unhealthy [sic]
- For a young woman age [sic] 18, what is the likelihood of developing high blood pressure?
- Has [sic] stress in college aged kids started an increase in hypertension in younger people [sic]

## Discussion

### Principal Results

Chatbots are growing in use across the internet, not only for consumer products and websites but also within health care settings. This paper described an exploratory study investigating how the design of a chatbot might impact its perceived trust, usability, and effectiveness in a health information search setting. The chatbot’s language was based on previous health care research that demonstrated that patients’ understanding of health information changes with language style and structure [4,18] as well as the cost of low health literacy on the health care system annually [7,8]. Chatbot persona was studied because it has been shown that other virtual agents may be more or less effective due to their perceived character [18]. Our results found that the chatbot’s responses which used technical language significantly increased the chatbot’s effectiveness but had no impact on trust or usability. The chatbot persona used in this study was found to significantly impact usability but had no impact on effectiveness or trust. Additionally, participants with higher health literacy reported higher trust in the chatbot. This finding is consistent with health literacy literature, which finds that people with higher health literacy generally have higher trust [33,34]. The qualitative transcript review revealed interesting insights about how people may use chatbots to gather health information and what they expect chatbots to be able to understand. The variation in sentence structure and grammar may be indicative of different subsets of users who interact with the chatbot, though that was not examined in this study. The use of shorthand is particularly interesting because it resembles more of a general, all-encompassing search pattern rather than a directed question-asking search pattern, perhaps indicative of those participants viewing the chatbot not as a person (as the persona looked to represent) but more as a search engine. Such generic searching demonstrates the need for chatbots to be able to process multiple kinds of search entries, whether it be formal input, shorthand, or all-encompassing search terms. These results show the potential that careful design may have on improving the effectiveness, usability, and trust in health care chatbots.

### Limitations and Future Work

A key limitation was the relative homogeneity of the participants within this study; participants were of similar ages (18-26 years) and education levels. Although this age range was selected to support a more homogeneous group of possible participants without direct experiences and knowledge associated with blood pressure, this does limit the generalizability of the study. Technical language responses may have been more effective because all of the participants were college students with relatively high health literacy, and thus, simplifying the responses may only have served as a detriment. In other populations with lower health literacy, nontechnical language may be more effective. Future work should more closely reflect the wider population ages, experiences, and health literacies in evaluating the usefulness of chatbots in health care applications. Additionally, future work should evaluate how the users’ identities and their intersectionality influence their interactions with chatbots to account for potential cultural and other biases that may be implemented in a chatbot’s design.

Health literacy and its impacts on chatbot language, trust, and usability need to be further studied. This study found that health literacy had an impact on the trust in the chatbot, which was to be expected based on previous research [33]. However, this study found that health literacy did not have an impact on usability, which is inconsistent with previous research [34]. Future research should use qualitative measures, such as interviews, to investigate why relationships or lack of relationships, such as language and effectiveness, health literacy and trust, or health and usability, are transpiring.

Another limitation is the simple persona used in this chatbot. This persona was not found to significantly impact effectiveness or trust. This may be because the persona used in this study was simple, and therefore, potentially unengaging; it included only a name and title, it did not have a picture or other visual stimuli, and it did not engage in any personalized dialogue (eg, asking the participant questions). This is supported in the qualitative transcript review, which found that most participants did not acknowledge Sarah (the chatbot’s persona), and few responded to the greeting, addressed Sarah at some other point in the dialogue, or thanked Sarah. Overall, most of the participants did not appear to engage with Sarah beyond its use as a chatbot to deliver information, suggesting that some participants used the chatbot as more of a conventional search engine rather than a conversational agent. Future studies should examine other ways of representing personas to evaluate whether personas in general are useful in this context. Other representations could include additional visual stimuli like pictures or avatar images. As the representations transform into 3D or virtual agents, the required characteristics need to change as well and follow other design patterns [18,35]. Additionally, this study examined only differences in the qualifications of the chatbot’s persona; further work should examine how larger differences in the persona’s identity may improve the chatbot’s effectiveness, usability, and trust. Given that the low health literacy portion of the US population is disproportionately represented by low-income or ethnic minority populations [11], personas that better reflect these minorities may aid in improving the chatbot’s effectiveness for these underrepresented groups. There may also be other user interface design strategies that better facilitate the effectiveness of chatbots for these groups.

Neither language nor persona had a significant effect on trust in our study. This could be in part due to trust being difficult to measure and quantify [36,37]. Trust is complex and dynamic with multiple factors contributing to an individual’s trust [38]. It is also possible that the participants in our study developed negative trust or conditional trust, where individuals expected the chatbot to fail at some point (ie, negative trust) but still reported trusting it or expected that the chatbot could do certain things or tasks in certain contexts (eg, focusing only on blood pressure information from a health care chatbot) and still reported trusting it (ie, conditional trust) [36]. An example of the negative trust may have occurred when even the 9 participants who received 5 or more responses of “I don’t know” to their questions still had relatively high trust. Other studies have shown that using different relational strategies (eg, small talk and empathic reactions) was not able to foster trust in a chatbot [39].

Lastly, although the experimental setting attempted to replicate a health care website with a chatbot, the setting was a static website with a simulated chatbot. The responses were not truly determined by an artificial agent but were instead accomplished with preconstructed responses resembling a messenger type system via a Wizard of Oz study. This replication may have impacted the results, as the responses were simulated by an experimenter and not by the technology. Since the responses were given by a person, there is a possibility for variability in how the experimenter responded. Along with the experimenter’s possible variability, there was variability in what questions participants asked and how participants asked those questions.

### Conclusions

With increased internet use in everyday life, the ways in which people obtain health care information are changing. It is important to continue to develop proper health care websites with information that can be personalized for users based on influential factors, such as age, gender, identity, and health literacy [5,8,40]. The ability of chatbots to provide personalized, private, and understandable health care information on a variety of topics makes it a promising tool, as health care trends toward web-based and remote processes. As participants look for health recommendations in different contexts and environments and with different devices and technologies, chatbots will need to be able to adapt to different needs. Understanding how those personal needs should change the language or presentation of the chatbot is crucial. Personalized health care information that is understood by each patient and caregiver will allow people to maintain ownership and have confidence in their health care decisions. As patients are better able to understand their health care needs, they can make decisions that allow for quicker recovery, create less impact on the health care system, and ultimately lower overall costs for the patient and the health care system.

Health care chatbots and telehealth medicine are also on the rise, not only in the last decade but particularly as a response to the COVID-19 pandemic. One technology implementation that saw an increase was telehealth medicine, where doctors and patients communicated virtually via videos, emails, and chats. Chatbots may be effective for these particular cases [41]. The COVID-19 pandemic additionally highlighted the global problem of health literacy disparity, as now more than ever people are forced to make health information–based decisions [42-44]. Therefore, an understanding of how to design and implement chatbots to effectively deliver health information is more crucial than ever. In order to develop effective design recommendations and guidelines for health care chatbots, future research needs to continue exploring how individuals perceive and interact with health care chatbots and their associated personas.

## Abbreviations

OR: odds ratio
PSSUQ: Post‐Study System Usability Questionnaire
